# Treatment Decisions in Patients With Potentially Nonsurvivable Burn Injury in Australia and New Zealand: A Registry-Based Study

**DOI:** 10.1093/jbcr/irac017

**Published:** 2022-02-16

**Authors:** Lincoln M Tracy, Michelle Gold, Sandra Reeder, Heather J Cleland

**Affiliations:** School of Public Health and Preventive Medicine, Monash University, Melbourne, Victoria, Australia; Palliative Care Service, Alfred Health, Melbourne, Victoria, Australia; School of Public Health and Preventive Medicine, Monash University, Melbourne, Victoria, Australia; Monash Partners Academic Health Science Centre, Clayton, Victoria, Australia; Victorian Adult Burns Service, Alfred Health, Melbourne, Australia

## Abstract

Whilst burn-related mortality is rare in high-income countries, there are unique features related to prognostication that make examination of decision-making practices important to explore. Compared to other kinds of trauma, burn patients (even those with nonsurvivable injuries) may be relatively stable after injury initially. Complications or patient comorbidity may make it clear later in the clinical trajectory that ongoing treatment is futile. Burn care clinicians are therefore required to make decisions regarding the withholding or withdrawal of treatment in patients with potentially nonsurvivable burn injury. There is yet to be a comprehensive investigation of treatment decision practices following burn injury in Australia and New Zealand. Data for patients admitted to specialist burn services between July 2009 and June 2020 were obtained from the Burns Registry of Australia and New Zealand. Patients were grouped according to treatment decision: palliative management, active treatment withdrawn, and active treatment until death. Predictors of treatment initiation and withholding or withdrawing treatment within 24 hours were assessed using multilevel mixed-effects logistic regression. Descriptive comparisons between treatment groups were made. Of the 32,186 patients meeting study inclusion criteria, 327 (1.0%) died prior to discharge. Fifty-six patients were treated initially with palliative intent and 227 patients had active treatment initiated and later withdrawn. Increasing age and burn size reduced the odds of having active treatment initiated. We demonstrate differences in demographic and injury severity characteristics as well as end of life decision-making timing between different treatment pathways pursued for patients who die in-hospital. Our next step into the decision-making process is to gain a greater understanding of the clinician’s perspective (eg, through surveys and/or interviews).

Burn injuries are a global issue, with the World Health Organization listing burns as the fourth most common form of trauma.^1^ Burn care has improved over recent decades, with real improvements in survival rates. Despite these advances, burn-related mortality remains a significant issue.^2,3^ On a global scale, burns account for an estimated 300,000 deaths each year; the majority of these deaths occurring in low- and middle-income countries.^4,5^ Burn-related mortality is lower in high-income countries. More than 7000 Australians and New Zealanders are hospitalized with a burn injury each year.^6,7^ Over half of these patients receive a higher level of care in a hospital with a specialist burn service.^8^ Approximately 1% of patients admitted to a specialist Australian or New Zealand burn service die as a consequence of their injuries each year.^8^ In 2019, the American Burn Association reported a 3% mortality rate for all cases over a 10-year period using data from the National Burn Repository.^9^ The main predictors of mortality are increasing severity of the burn (usually indicated by percent of TBSA burned), increasing age, and the presence of an inhalation injury.^10,11^ Gender is also likely to be a predictor of mortality following burn injury.^12^

There is only a small pool of literature regarding primary or specialist palliative care for burns patients that focuses primarily on interventions for physical symptoms.^13^ A recent scoping review from Bayuo et al concluded that while palliative care is applicable in burns management, its current role is mostly confined to the end of life period (ie, the last few days of life), suggesting a lack of integration with the broader management process.^14^ In particular, palliative care would be appropriate in cases where the initial prognosis may be poor but active treatment will be commenced (eg, trial of life therapy). Support would be provided to the patient, their family, and staff members to envisage potential outcomes if treatments proved unsuccessful, or how to decide when to opt for a comfort-based approach, for example. Previous studies have identified both objective and subjective criteria (eg, injury severity, preexisting comorbidities, clinician knowledge, and previous experience, etc.) that contribute to the treatment decision-making process.^15–17^ Research in international settings regarding the timing of decision-making to withhold (not deliver) or withdraw (cease delivery of) active (ie, life-sustaining) treatment has also been undertaken.^18,19^ Mahar et al investigated clinical aspects of patients that die whose injuries were considered either survivable or nonsurvivable at admission at a single site within the Australian state of Victoria.^16^ Other studies have explored predictors of withdrawing active treatment following burn injury.^20,21^

However, there is yet to be a larger-scale investigation of treatment decision-making in patients with potentially nonsurvivable burn injury in Australia and New Zealand. Therefore, the aims of this study were to 1) investigate the frequency of decisions to withhold or withdraw treatment following potentially nonsurvivable burn injury in Australian and New Zealand burn services; 2) identify predictors of initiating active treatment in patients with potentially nonsurvivable burn injury; 3) compare the demographic and injury event characteristics, in-hospital management, and timing of decision-making for patients in different treatment decision groups; and 4) identify predictors of withholding or withdrawing active treatment within 24 hours of admission.

## METHODS

### Setting

This study used data from the Burns Registry of Australia and New Zealand (BRANZ) for admissions to specialist burn services between July 1, 2009 and June 30, 2020. The BRANZ is an international clinical quality registry that has collected epidemiological, quality of care, and in-hospital outcome data for patients admitted to specialist burn services in Australia and New Zealand since July 2009. Approximately 3000 admissions to specialist burn services are collected by the BRANZ each reporting year. Further information about the registry (including inclusion and exclusion criteria) has been published elsewhere.^22–25^ During the 11-year study period, the following services contributed data to the BRANZ: Alfred Hospital, Fiona Stanley Hospital, Middlemore Hospital, Royal Adelaide Hospital, Royal Darwin Hospital, Royal Children’s Hospital, Women’s & Children’s Hospital, Perth Children’s Hospital, Royal Brisbane & Women’s Hospital, Queensland Children’s Hospital, Royal North Shore Hospital, Children’s Hospital at Westmead, Concord Repatriation General Hospital, Royal Hobart Hospital, Townsville University Hospital, Christchurch Hospital, Waikato Hospital, and Hutt Hospital.

### Inclusion and Exclusion Criteria

We focused on patients who died during their first admission as entered into the BRANZ for the majority of analyses; all patients with data on the treatment decision field were included in identifying predictors of active treatment. Patients were excluded if their date of injury was missing or unknown, or if their discharge disposition was missing, not stated, or inadequately described. Patients where data were missing or not stated for the treatment decision field (ie, to determine, for patients who died, whether the medical decision was to withdraw or withhold medical treatment) were counted in the overall number of patients who died but were excluded from further analyses.

### Data Management

In line with our aims, patients were grouped according to treatment decision: *palliative management* (ie, burn was assessed as nonsurvivable, the goals of care were considered to be palliative, and the patient received end of life care on admission), *active treatment started then withdrawn* (ie, active treatment initiated but subsequently withdrawn as further treatment was considered nonsurvivable), and *active treatment until death* (ie, active treatment was initiated [not withheld] and continued until the time of death [not withdrawn]). We defined active treatment as undergoing excisional debridement as part of a burn wound management procedure in theater or the initiation of enteral feeding or venous thromboembolism prophylaxis.

Age at the time of injury was calculated using date of birth and date of injury data and presented both as a continuous and dichotomous variable (<65 and ≥65 years). Several demographic, injury event, injury severity, and in-hospital outcomes variables were dichotomized: male gender, primary injury cause (flame burns = 1, all other causes = 0), location where the burn injury occurred (home or usual residence = 1, all other locations = 0), whether there was documented evidence of an inhalation injury, whether the patient sustained a full thickness burn, whether the patient underwent a burn wound management procedure in theater, whether the patient was admitted to the intensive care unit (ICU), and whether the patient was placed on a mechanical ventilator. Injury intent was coded into two dichotomous variables to identify unintentional injuries and intentional self-harm injuries. The TBSA burned served as the primary measure of injury severity. The continuous TBSA value was also categorized as follows: 0% to 9%, 10% to 19%, 20% to 49%, 50% to 79%, and 80% to 100%. Major burns were defined as burns equal to or greater than 20% TBSA. The comorbid status of patients was defined using the Charlson Comorbidity Index (CCI),^26^ mapped from International Statistical Classification of Diseases and Related Health Problems, Tenth Revision, Australian Modification (ICD-10-AM) codes.^27^ Patients with ICD-10-AM codes accompanying their admissions data were grouped according to CCI weight (0 and ≥1).

Time-to-event data (ie, time from injury to admission, time from admission to decision, time from decision to death) was calculated using dates and times of injury, admission, treatment decision, and death. Time from admission to decision was dichotomized as within or beyond 24 hours as per Bartley et al^20^ and categorized as less than 24 hours, between 1 and 3 days, between 4 and 6 days, between 7 and 13 days, between 14 and 20 days, and beyond 21 days. Time from decision to death was categorized as less than 24 hours, between 1 and 3 days, between 4 and 6 days, and 7 days and beyond. Time to death was categorized as less than 24 hours, between 1 and 3 days, between 4 and 6 days, between 7 and 13 days, between 14 and 20 days, between 21 and 27 days, and beyond 28 days as per Swanson et al.^28^

### Statistical Analysis

Demographic, injury event and severity, comorbidity, in-hospital management (eg, surgical procedures, ICU admissions), and time-to-event data were described using summary statistics (frequencies and percentages for categorical variables, medians and interquartile ranges [IQRs] or mean and *SD* for continuous variables). Data were compared between groups using chi-square tests (for categorical variables) and Kruskal–Wallis tests or one-way analysis of variance for continuous variables. To estimate the predictors of active treatment initiation and the decision to withhold or withdraw active treatment within 24 hours of admission, we used multilevel mixed-effects logistic regression models controlling for the random effects of the contributing service and the fixed effects of covariates known to affect the outcomes: age, gender, primary cause of injury, intent, %TBSA burned, presence of an inhalation injury, and presence of a full thickness burn.^20,21^ Univariate models were run first. Predictors displaying an association with active treatment initiation (ie, *P* < .20) were included in the multivariable model, for which adjusted odds ratios (ORs) and 95% confidence intervals (CIs) are reported. Sensitivity analyses were conducted to investigate the effect of including CCI weight in the regression model (ie, limiting the regression to the cohort of patients with ICD-10-AM codes accompanying their admission data). All data management and statistical analyses were performed using Stata Version 14 (StataCorp, College Station, TX). Figures were produced in the R statistical environment version 4.0.3^29^ using the *tidyverse*^30^ package.

### Ethics Approval

Ethics approval for this project was granted by the Monash University Human Research Ethics Committee (project number 29838).

## RESULTS

### Sample Description

There were 32,186 patients with an acute admission captured by the BRANZ during the 11-year study period (Supplementary Figure 1). Of these, 358 patients (1.1%) died prior to discharge. The proportion of patients who died each year changed during the study (*P* = .043, Supplementary Table 1). Treatment decision data (ie, to determine whether the medical decision was to withdraw or withhold medical treatment) were missing for 29 patients, leaving a final sample of 327 (1.0% of total admissions). For the patients where treatment decision data were recorded, 56 patients (17.1%) received palliative management on admission, 227 patients (69.4%) received active treatment initially that was withdrawn prior to their death, and 44 patients (13.5%) received active treatment until the time of their death. The most commonly described cause of death in the palliative management group was burns shock (n = 26, 50.0%), while multisystem organ failure was the most common cause of death in the two active treatment groups (n = 84, 38.5% for active treatment withdrawn group; n = 18, 46.2% for active treatment until death group). Multisystem organ failure was the second most common cause of death in the palliative management group (n = 14, 26.9%). Burns shock was the second most common cause of death in the active treatment started then withdrawn group (n = 48, 22.0%). Cardiac-related incidents (eg, acute myocardial infarction) were the second most common cause of death in the active treatment until death group (n = 8, 20.5%). Sepsis and pulmonary-related causes of death (eg, pulmonary embolism, pneumonia) occurred less frequently, with the greatest proportion of these cases occurring in the active treatment initiated then withdrawn group.

### Predictors of Initiating Active Treatment

Age, gender, primary cause of injury, intent, %TBSA burned, presence of an inhalation injury, and presence of a full thickness burn were all univariate predictors of initiating active treatment and entered into the multivariable model (Table 1). There were reduced odds for initiating active treatment with each increasing year of age (adjusted OR [aOR] 0.93, 95% CI 0.92–0.95). There were reduced odds of initiating active treatment with increasing %TBSA burned (aOR 0.94, 95% CI 0.93–0.96). Limiting the analyses to patients with ICD-10-AM codes (to examine the effect of including comorbidities in the model) accompanying their admissions data also identified patients with full thickness burns had reduced odds of initiating active treatment (Supplementary Table 2).

**Table 1.** Predictors of initiating active treatment

	OR (95% CI)	*P*	aOR (95% CI)	*P*
Age	0.96 (0.94–0.97)	<.001	0.93 (0.91–0.95)	<.001
Male		.03		.07
No (reference)	1.00		1.00	
Yes	1.83 (1.08–3.12)		1.90 (0.96–3.79)	
Flame burn		<.001		.07
No (reference)	1.00		1.00	
Yes	0.06 (0.03–0.16)		0.33 (0.10–1.10)	
Unintentional injury		<.001		.31
No (reference)	1.00		1.00	
Yes	14.49 (8.50–24.70)		1.49 (0.69–3.24)	
%TBSA burned	0.92 (0.92–0.93)	<.001	0.93 (0.92–0.95)	<.001
Inhalation injury		<.001		.09
No (reference)	1.00		1.00	
Yes	0.03 (0.01–0.05)		0.48 (0.21–1.11)	
Full thickness burn		<.01		.10
No (reference)	1.00		1.00	
Yes	0.08 (0.04–0.16)		0.47 (0.19–1.19)	

*aOR*, adjusted odds ratio; *CI*, confidence interval; *OR*, odds ratio.

### Demographic and Injury Event Characteristics

Table 2 compares the demographic and injury event characteristics of the three treatment groups for patients who died during their admission. The median (IQR) age of patients was 60 (42–76) years, but there was no difference in age between treatment groups. The sample was predominantly male (n = 202, 61.8%), which reflects the typical gender mix seen within the BRANZ. Eighty percent of patients who received active treatment until death were male, while 57% of patients who received palliative management on arrival were male. More than three quarters of patients (n = 254, 77.7%) sustained a flame burn, while more than two thirds of patients (n = 219, 70.0%) sustained their burn at home. A greater proportion of patients who received active treatment until their death had injuries arising through unintentional means, while a greater proportion of patients who received palliative management on arrival were documented to have intentionally self-harmed. Over half the patients who died in-hospital (n = 179, 55.8%) had documented evidence of an inhalation injury in addition to their cutaneous burn. Three quarters of patients (n = 218, 74.7%) sustained a full thickness burn. The median (IQR) TBSA burned was 48.5% (16.0–79.0) and did not differ between the three treatment groups. Of the patients who received palliative management on arrival, one eighth of patients (n = 7, 12.5%) sustained a burn <20% TBSA. Five patients with a burn exceeding 80% TBSA received active treatment until their death. More than three quarters of patients (n = 256, 78.3%) had ICD-10-AM codes accompanying their admissions data. A greater proportion of patients who received palliative management on arrival had a CCI weight of 0 (indicating no comorbidities), while a greater proportion of patients who received active treatment until their death had a CCI weight ≥1.

**Table 2.** Demographic, event, and injury severity characteristics by treatment decision group

	Palliative Management	Active Treatment Started Then Withdrawn	Active Treatment Until Death	*P*
N	56	227	44	
Age, median (IQR), years	57.0 (39.5, 78.5)	61.0 (44.0, 76.0)	58.5 (42.5, 70.5)	.68
Age group				.70
<65 years	32 (57.1%)	129 (56.8%)	28 (63.6%)	
≥65 years	24 (42.9%)	98 (43.2%)	16 (36.4%)	
Male	32 (57.1%)	135 (59.5%)	35 (79.5%)	.032
Flame burn	51 (91.1%)	166 (73.1%)	37 (84.1%)	.008
Sustained burn at home^a^	32 (59.3%)	164 (75.2%)	23 (56.1%)	.008
Unintentional injury^b^	27 (48.2%)	136 (61.3%)	36 (83.7%)	.001
Intentional self-harm^c^	22 (39.3%)	56 (25.2%)	5 (11.6%)	.007
Inhalation injury^d^	38 (70.4%)	119 (52.7%)	22 (53.7%)	.060
Full thickness burn^e^	43 (81.1%)	148 (72.2%)	27 (79.4%)	.33
TBSA, median (IQR)%^f^	78.5 (34.0, 93.5)	42.0 (14.5, 70.0)	38.0 (10.2, 62.0)	<.001
TBSA group^f^				<.001
0%–9%	6 (10.7%)	37 (16.6%)	9 (20.9%)	
10%–19%	< 5	29 (13.0%)	7 (16.3%)	
20%–49%	13 (23.2%)	52 (23.3%)	7 (16.3%)	
50%–79%	8 (14.3%)	58 (26.0%)	15 (34.9%)	
80%–100%	28 (50.0%)	47 (21.1%)	5 (11.6%)	
ICD codes entered	45 (80.4%)	181 (79.7%)	30 (68.2%)	.22
CCI weight*				<.001
0	34 (75.6%)	84 (46.4%)	10 (33.3%)	
≥1	11 (24.4%)	97 (53.6%)	20 (66.7%)	

Data presented as frequency (percentage) unless otherwise specified.

*CCI*, Charlson Comorbidity Index; *ICD*, International Statistical Classification of Diseases and Related Health Problems, Tenth Revision, Australian Modification; *IQR*, interquartile range; *N*, number.

Data missing for

^a^14 patients,

^b^6 patients,

^c^6patients,

^d^6patients,

^e^35 patients, and

^f^5 patients.

*For patients with ICD codes entered.

### In-hospital Management

Table 3 compares the in-hospital management of the three different treatment groups. A greater proportion of patients who received active treatment until their death underwent a burn wound management procedure in theater, compared to patients who had active treatment withdrawn or withheld. More than 80% of patients (n = 266, 81.4%) were admitted to the ICU during their admission. A greater proportion of patients who had active treatment initiated and then withdrawn were admitted to the ICU compared to the remaining two treatment groups. For patients admitted to the ICU, patients who received active treatment until their death had the longest ICU stay (median [IQR] 174.1 [78.5–473.9] hours), while patients who received palliative management on admission had the shortest ICU stay (10.9 [5.8–21.9] hours). Ninety-three percent (n = 247) of patients admitted to the ICU were mechanically ventilated.

**Table 3.** In-hospital management data by treatment decision group

	Palliative Management	Active Treatment Started Then Withdrawn	Active Treatment Until Death	*P*
N	56	227	44	
Burn wound management in theater^a^	< 5	123 (54.2%)	34 (79.1%)	<.001
ICU admission	40 (71.4%)	195 (85.9%)	31 (70.5%)	.006
ICU LOS hours, median (IQR) hours*^,^^b^	10.9 (5.8, 21.9)	91.7 (18.2, 297.0)	174.1 (78.5, 473.9)	<.001
Mechanical ventilation*	36 (90.0%)	180 (92.3%)	31 (100.0%)	.23
Mechanical ventilation, median (IQR) hours†,^c^	8.0 (3.0, 13.5)	54.0 (12.0, 215.9)	174.1 (48.5, 358.5)	<.001

Data presented as frequency (percentage) unless otherwise specified.

*ICU*, intensive care unit; *IQR*, interquartile range; *LOS*, length of stay; *N*, number.

Data missing for

^a^2 patients,

^b^3 patients, and

^c^55 patients.

*For patients admitted to the ICU.

^†^For patients who received mechanical ventilation in the ICU.

### Time-Based Events

**Figure 1.**
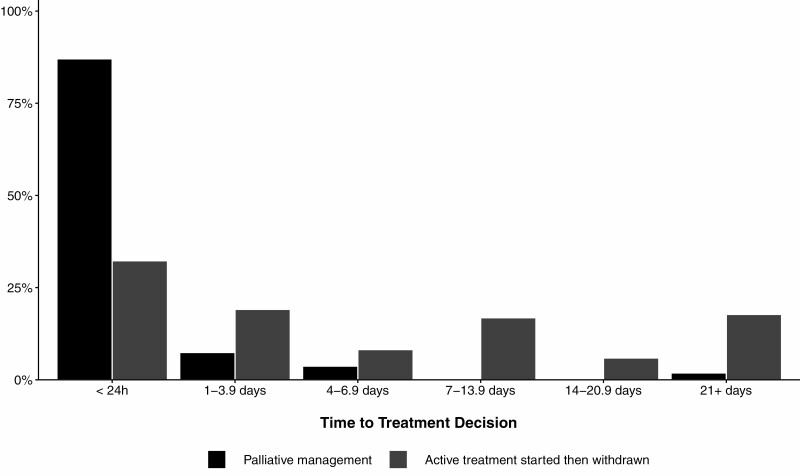
Time to decision for patients where treatment with withheld or withdrawn. Percentage is relative to total number of participants in each treatment group.

**Figure 2.**
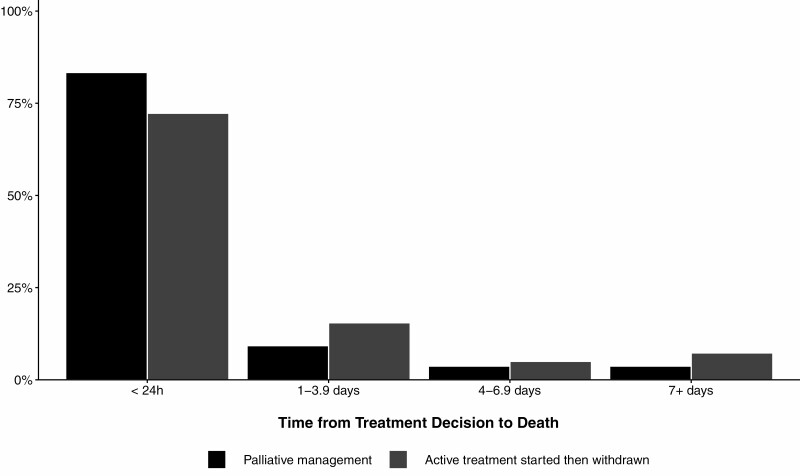
Time from decision to death in patients where treatment with withheld or withdrawn. Percentage is relative to total number of participants in each treatment group.

**Figure 3.**
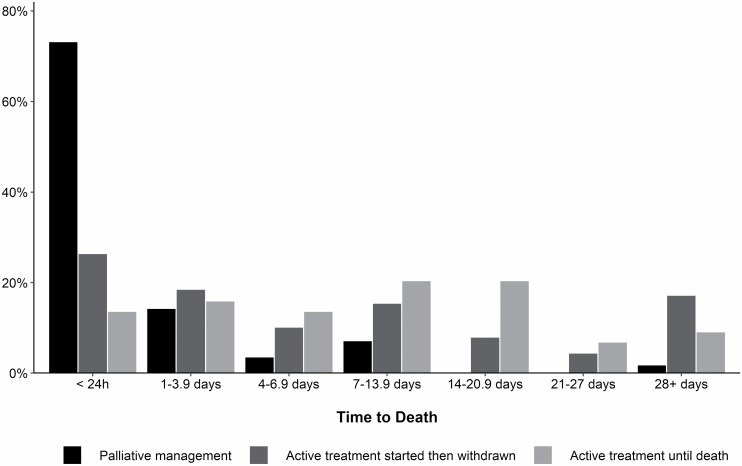
Time to death (from admission) for the three treatment decision groups.

The median (IQR) time from injury to admission at a specialist burn service was 4.7 (2.1–11.6) hours. Patients who received palliative management had a shorter median time to admission compared to patients who received active treatment until death (Table 4). The median time from admission to treatment decision was shorter for patients who received palliative management (3.4 [0.8–11.2] hours) compared to those who had active treatment initiated then withdrawn (87.3 [14.2–327.0] hours). A greater proportion of decisions to follow a palliative approach (ie, withhold active treatment) than withdraw treatment occurred in the first 24 hours after admission (*P* < .001, Figure 1). After the treatment decision was made, patients who had treatment withdrawn (5.3 [1.0–30.0] hours) had a shorter median time to death than patients where treatment was withheld (7.0 [2.8–14.9] hours). The proportion of deaths within 24 hours after a decision to pursue a palliative approach was made did not differ between groups (*P* = .10, Figure 2). Patients who received active treatment until the time of their death had a longer median hospital length of stay (LOS; 7.5 [2.3–19.1] days) compared to patients in the other two treatment groups (5.7 [0.9–16.4] days for active treatment withdrawn, 0.5 [0.3–1.0] days for palliative management). A greater proportion of patients who received palliative management died within 24 hours of admission compared to the two patient cohorts who initiated active treatment (*P* < .001, Figure 3). Thirteen percent of patients who received palliative management from the outset had a treatment decision recorded more than 24 hours after admission. Thirteen percent of all patients died more than 28 days after admission.

**Table 4.** Time-to-event data by treatment decision group

	Palliative Management	Active Treatment Started Then Withdrawn	Active Treatment Until Death	*P*
N	56	227	44	
Time from injury to admission, median (IQR) hours^a^	3.2 (1.7, 6.4)	4.7 (2.1, 12.6)	7.1 (3.0, 20.5)	.017
Time from admission to decision, median (IQR) hours^b^	3.4 (0.8, 11.2)	87.3 (14.2, 327.0)	—	<.001
Time from decision to death, median (IQR) hours^c^	7.0 (2.8, 14.9)	5.3 (1.0, 30.0)	—	.63
Hospital LOS, median (IQR) days	0.5 (0.3, 1.0)	5.7 (0.9, 16.4)	7.5 (2.3, 19.1)	<.001

*IQR*, interquartile range; *LOS*, length of stay; *N*, number.

Data missing for

^a^3 patients,

^b^9 patients, and

^c^9 patients.

### Predictors of Decision to Withhold or Withdraw Active Treatment Within 24 Hours

Age, gender, primary cause of injury, intent, %TBSA burned, presence of an inhalation injury, and presence of a full thickness burn were all univariate predictors of withholding or withdrawing active treatment within 24 hours and entered into the multivariable model (Supplementary Table 3). Only increasing age (aOR = 1.02, 95% CI 1.00–1.04), increasing %TBSA burned (aOR = 1.04, 95% CI 1.03–1.06), and the presence of an inhalation injury (aOR = 2.22, 95% CI 1.06–4.63) were predictive of the decision to withhold or withdraw active treatment within 24 hours.

## DISCUSSION

This study investigated the frequency and timing of treatment decisions for patients with potentially nonsurvivable burn injuries in specialist burn services, as well as exploring the differences in patient and injury characteristics between patients in the three different treatment approaches. In-hospital mortality was low; approximately 1% of patients died during their admission. Of the patients who died, more than two-thirds had active treatment initiated and withdrawn at a later stage. Increasing age and increasing %TBSA burned were associated with reduced odds of having active treatment initiated. The decision to withhold active treatment was made more quickly than the decision to withdraw active treatment once it had been initiated. Most patients died within 24 hours after the decision to withhold or withdraw medical treatment was made.

With increasing capacity to deliver care that results in survival, the issue of quality of life has become more significant in considering treatment decisions for patients with potentially nonsurvivable injuries. Death following burn injury is typically delayed, especially in the absence of inhalation injury and for those patients who reach hospital,^28^ and there are critical treatment decisions to be made in the immediate postinjury phase and beyond. A key component of the *immediate* decision-making process is determining whether the burn is survivable or not. This decisions will determine whether active treatment or palliative management is initiated. Palliative care aims to improve the quality of life of patients (and their families) with incurable disease through early identification and the treatment of physical symptoms and other issues. Palliative care originated in the end-stage cancer care setting, and the general principles of palliative care in an oncological setting are broadly applicable to decision-making and delivery of end of life burn care.^31,32^ However, limitations on the applicability of oncological palliative care-focused literature in burns patients have been noted^33^ as burns patients usually die within hours or days once their injuries are deemed nonsurvivable.^28,34^ Benefits from integrating palliative care in burn services have been identified.^35^ The International Society for Burn Injuries has recommended that relief from pain/anxiety and emotional support of the patient should be provided once the injury is considered nonsurvivable.^36^

The overall in-hospital mortality rate for the current study was 1.1%. Our observation that more than two-thirds of the patients who died received some form of active treatment which was later withdrawn is somewhat consistent with previous literature. This figure is higher than the 33% of patients reported by Dokter et al in their review of deaths in two Dutch burn centers between 2006 and 2011.^37^ However, it is lower than Pham et al’s report that 84% of 126 patients who died within 72 hours of admission to the University of Washington Regional Burn Centre between 1995 and 2007 had life-sustaining treatment withdrawn prior to death.^17^ Less than 15% of patients in the current study received active treatment until death. This is lower than the 31.3% reported by Bartley et al^20^ and the 35.2% reported by Dokter et al^37^ but similar to the 16% reported by Pham et al.^17^ This variation may be explained by differences in patient casemix, differences between different burn services and countries with respect to end of life decision-making protocols and procedures, and differences in study population and data management (eg, Pham et al only looked at deaths within 72 hours of admission, Bartley et al excluded patients who died despite receiving active treatment until death, etc.).

This study identified that increasing age and TBSA burned were associated with reduced odds of having active treatment initiated upon arrival to a specialist burn service. These findings are generally consistent with a previous study of Sheckter et al, who sought to evaluate predictors of palliative care service utilization in their sample of burns patients from the Nationwide Inpatient Sample.^21^ Sheckter et al reported that increasing age and %TBSA burned, full thickness burns, and inhalation injury predicted increased odds of palliative care utilization. These results are also somewhat consistent with Bartley et al, who reported that female gender, increasing TBSA burned, increasing age, and inhalation injury were predictors of withdrawal of life-sustaining treatment.^20^ All three studies are consistent with previous findings suggesting that increasing age and burn size are associated with mortality following burn injury.^10–12^ This implies that these elements contribute strongly to burn care clinician’s assessments of survival likelihood and end of life decision-making processes.

Differences in demographic and injury severity characteristics were observed between treatment groups. Comparisons with other studies are difficult, previous research in this context varies with respect to inclusion/exclusion criteria and data management.^15–17,19–21,28,37^ Our observation that the proportion of male patients differed between treatment groups is in contrast to that of Dokter et al, who reported no association between treatment decision and gender.^37^ The reason for this inconsistency is unclear, as male patients outnumber female patients approximately 2:1 in both Australia/New Zealand and the Netherlands.^8,38^ In contrast, flame burns were the predominant cause of injury across all three treatment groups in both studies. Anecdotal evidence suggests that injury intent may play a role in the decision to withhold or withdraw treatment. The current data do not support this anecdotal evidence, with no observed association between injury intent and the initiation of active treatment after accounting for other relevant factors. Intentional burn injuries have been reported to affect a greater percentage of TBSA,^8,39^ but the association with increased odds of mortality has been debated.^39^ Further research into how injury intent affects end of life decision-making processes is required. Our finding that a smaller proportion of patients who received palliative management from the outset of their admission had a CCI weight ≥1 (compared to the other two treatment groups) is counterintuitive and should be investigated in greater detail in future studies. The finding that a greater proportion of patients who received some form of active treatment underwent a burn wound management procedure in theater may more commonly involve substantial treatment measures such as excisional debridement or grafting. However, for patients who received palliative management, such trips to theater may simply involve scrubbing and dressing wounds or other similar procedures to minimize distress to family and loved ones who attend the patient.

The finding that the majority of decisions to cease (or not pursue) active treatment were made within 24 hours of admission is consistent with previously published literature.^16,19^ In contrast, the timing of the decision to withdraw previously initiated active treatment was more variable. The decision to withdraw active treatment was made more than a week after admission for more than 40% of patients in the current sample. Reasons for withdrawal of treatment are variable: some patients may receive active treatment for several days until a next of kin can be contacted and their treatment wishes ascertained. Recent data from Ota et al support this notion; patients without a next of kin are less frequently transitioned to palliative care compared to patients with a next of kin.^40^ When there is no clear indication on presentation that an injury is nonsurvivable, clinicians will instigate an active treatment approach. Some patients will respond to and improve with this treatment whereas others will deteriorate in spite of the active treatment or develop new complications, and further treatment may be deemed futile.

Our finding that patients who received active treatment until death had a longer hospital length of stay (or time to death) than patients who had treatment withheld or had treatment withdrawn is consistent with previous research.^17,37^ This may reflect the considerable resources available for critical care life support, and reinforces the need for active and timely decision-making if burdensome futile treatment is to be avoided. The distribution of the time to death across patient groups was also consistent with Swanson et al, where more than 50% of patients died within the first 72 hours.^28^ Increasing age, increasing %TBSA burned, and inhalation injury were predictive of the decision to withhold or withdraw active treatment within 24 hours. This is also consistent with a previous study from Partain et al, who observed that the magnitude of injury (ie, Baux score; age + %TBSA burned) was the primary driver underlying clinician decision-making^19^ and Dokter et al, who concluded that the decision to withhold active treatment is mostly influenced by %TBSA burned.^37^ However, it is important to note that Partain et al focused specifically on geriatric patients and only performed descriptive analyses, which may explain some of the inconsistencies between the current and previous findings.^19^

The risk of in-hospital mortality is relatively well understood and universally considered in burns patients. By characterizing the broad patterns and timings of decision-making and death in our registry-based cohort, this study forms the basis for further investigation into ethical considerations and approaches that underpin the end of life decision-making processes required for patients with nonsurvivable injuries. Such research requires looking beyond data collected in large clinical quality registries (or potentially even medical records) and exploring the underlying thought processes of clinicians responsible for these decisions. Studies continuing this line of research utilizing survey and interview approaches are underway; findings will be published elsewhere. There is currently relatively little detailed consideration of these matters in the burns literature and the lack of an explicit consensus regarding relevant factors and key points in the process potentially provides little support for individuals seeking to make sound decisions in individual situations that are (fortunately) very uncommon.

However, the existing literature and the current study suggest burn clinicians may rely on more established mortality predictors to guide treatment decisions for patients with potentially nonsurvivable injuries. This contrasts with decision-making in malignant hematology or oncology settings, where decision-making processes are more clinician- and/or patient-dependent.^41^ This may be because definitive data regarding the “tipping point” for stopping blood transfusions in advanced myeloma or another course of antibiotics/noninvasive ventilation for patients with end-stage chronic obstructive pulmonary disease is lacking compared to end of life decision-making in burns patients. Despite the overall indication that burn treatment decisions are broadly made with objective predictors of outcome, it is recognized that patient preferences and other individual factors are relevant considerations in many treatment decisions. It is not possible to ascertain a complete understanding of the factors that dictate withholding treatment from the outset or withdrawing previously initiated treatment from the current study alone. It is hoped that the aforementioned additional research addresses some of the unanswered questions.

The limitations of this study must be addressed. Unlike Mahar et al^16^ or Pham et al,^17^ the BRANZ does not have access to narrative notes from clinicians treating patients with potentially nonsurvivable burn injury. Consequently, we were unable to further investigate the rationales for the decision to withhold or withdraw active treatment in our cohort of patients or determine whether the palliative process was managed by the primary burn care team or another dedicated service (eg, palliative care, ICU, etc.). As a result, it was not possible to explore the influence of additional factors (eg, patient, family, or surrogate wishes, etc.) beyond the demographic and injury severity/event characteristics captured by the BRANZ. In addition, the different services contributing to the BRANZ may utilize different protocols or tools in end of life decision-making processes. This may have influenced the results of the current study if services are at the extremes of either withholding active treatment or providing active treatment until death. Finally, as we analyzed data from specialist burn services in Australia and New Zealand, these results may not necessarily translate to other settings.

## CONCLUSIONS

This study shows that although death in burns patients occurs via the three broad pathways considered here, the majority of patients received some form of active treatment that was later withdrawn. Patients treated with palliative intent from the time of admission had larger burns than patients who had active treatment initiated. Predictors of initiating and withholding or withdrawing active treatment (increasing age and %TBSA) were identified. Withholding treatment in certain cases may not be a difficult clinical decision but may be emotionally challenging. The proportion of patients who receive active treatment for some time before it is withdrawn, or who die while receiving active treatment, suggest specialist clinicians may need to consider many factors in their decision-making process to help determine which treatment pathway is pursued.

## Supplementary Material

irac017_suppl_Supplementary_Material

## Notes

Data Availability Data from the BRANZ are not publically available. Access to BRANZ data requires ethics approval from an institutional human research ethics committee. Further information about requesting data from the BRANZ is available online: https://www.monash.edu/medicine/sphpm/branz/data-requests.

Funding: This work was supported by the Bethlehem Griffiths Research Foundation [BGR2108]. The BRANZ has received funding from the Australian and New Zealand Burn Association, the Australian Commission on Safety and Quality in Health Care (2008–2009), the Julian Burton Burns Trust (2008–2013), the Helen Macpherson Smith Trust (2010–2012), the Thyne Reid Foundation (2011–2013), the Australasian Foundation for Plastic Surgery (2013–2017), the New Zealand Accident Compensation Corporation (2013–2020), the Clipsal by Schnieder Electric National Community Grants Program (2017), the HCF Research Foundation (2018–2019), and the Victorian Agency for Health Information (2020–2021). Individual burn services also contribute funding to the BRANZ to ensure its ongoing sustainability. The funding sources played no role in study design, the collection, analysis, and interpretation of data, in the writing of the report, or the decision to submit the article for publication.

Portions of this work were presented at the 44th Annual Scientific Meeting of the Australian and New Zealand Burn Association.
