# Supplementary material for: Treatment Decisions in Patients With Potentially Nonsurvivable Burn Injury in Australia and New Zealand: A Registry-Based Study
Source: J Burn Care Res. 2022 Feb 16;44(3):675–84. doi: 10.1093/jbcr/irac017 (PMC10152993; doi:10.1093/jbcr/irac017)
Supplement: irac017_suppl_Supplementary_Material [file irac017_suppl_supplementary_material.docx]

**Supplementary Materials**

**
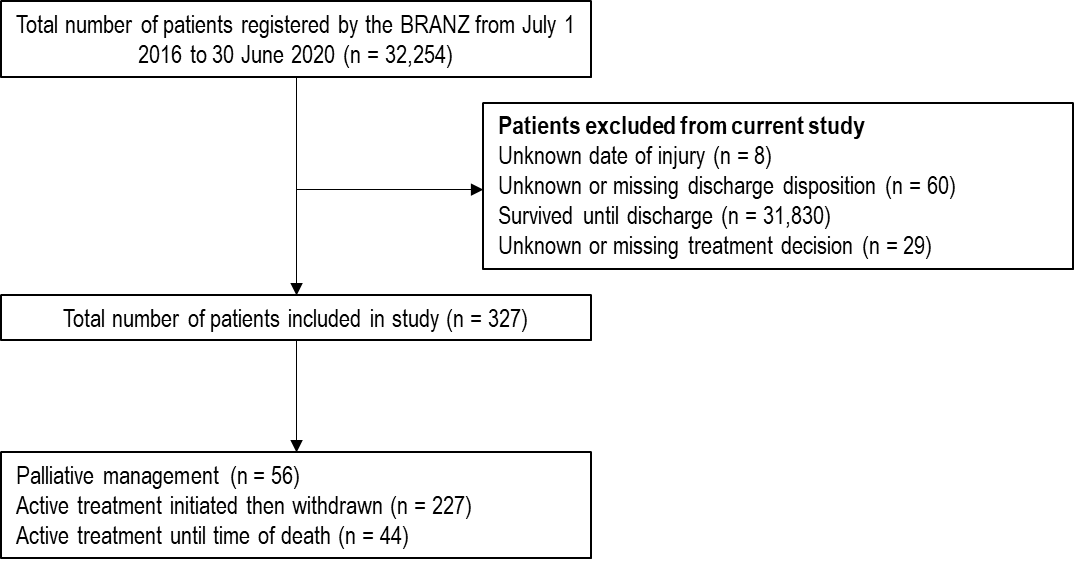
**

**Supplementary Figure 1.** Patient flow chart. BRANZ = Burns Registry of Australia and New Zealand.

| **Supplementary Table 1 – Age and TBSA of burn patients between 2009/10 and 2019/20** | | | | |
| --- | --- | --- | --- | --- |
| **Period** | **Admissions** | **Deaths†** | **Age‡** | **TBSA‡** |
| 2009/10 | 2,170 | 35 (9.8%) | 30.7 (22.7) | 7.3 (11.6) |
| 2010/11 | 2,484 | 19 (5.3%) | 28.7 (22.2) | 6.6 (9.7) |
| 2011/12 | 2,807 | 31 (8.7%) | 29.7 (23.3) | 6.4 (10.1) |
| 2012/13 | 2,772 | 36 (10.1%) | 29.6 (23.0) | 6.5 (10.1) |
| 2013/14 | 2,837 | 29 (8.1%) | 28.2 (23.2) | 5.7 (8.8) |
| 2014/15 | 2,771 | 23 (6.5%) | 28.6 (23.8) | 6.0 (9.4) |
| 2015/16 | 2,883 | 36 (10.1%) | 29.7 (23.1) | 6.3 (10.6) |
| 2016/17 | 3,314 | 50 (14.0%) | 31.1 (23.7) | 6.1 (10.7) |
| 2017/18 | 3,463 | 36 (10.1%) | 31.7 (23.3) | 6.0 (10.3) |
| 2018/19 | 3,346 | 29 (8.2%) | 32.3 (23.4) | 5.6 (9.2) |
| 2019/20 | 3,339 | 32 (9.0%) | 32.6 (23.2) | 6.1 (9.9) |
| Total | 32,186 | 356 (100%) | 30.4 (23.3) | 6.2 (10.0) |
| TBSA = total body surface area.  † Data presented as frequency (percentage).  ‡ Data presented as mean (standard deviation). | | | | |

| **Supplementary Table 2. Predictors of initiating active treatment** | | | | |
| --- | --- | --- | --- | --- |
|  | **OR (95% CI)** | ***p-*value** | **aOR (95% CI)** | ***p-*value** |
| Age | 0.95 (0.94-0.96) | < 0.001 | 0.91 (0.89-0.94) | < 0.001 |
| Male |  | 0.02 |  | 0.10 |
| No (reference) | 1.00 |  | 1.00 |  |
| Yes | 1.99 (1.10-3.60) |  | 1.95 (0.88-4.34) |  |
| Flame burn |  | < 0.001 |  | 0.33 |
| No (reference) | 1.00 |  | 1.00 |  |
| Yes | 0.07 (0.02-0.19) |  | 0.52 (0.14-1.92) |  |
| Unintentional injury |  | < 0.001 |  | 0.41 |
| No (reference) | 1.00 |  | 1.00 |  |
| Yes | 16.24 (8.92-29.58) |  | 1.48 (0.58-3.76) |  |
| %TBSA burned | 0.92 (0.91-0.93) | < 0.001 | 0.93 (0.91-0.94) | < 0.001 |
| Inhalation injury |  | < 0.001 |  | 0.10 |
| No (reference) | 1.00 |  | 1.00 |  |
| Yes | 0.02 (0.01-0.05) |  | 0.43 (0.16-0.1.18) |  |
| Full thickness burn |  | < 0.001 |  | 0.02 |
| No (reference) | 1.00 |  | 1.00 |  |
| Yes | 0.07 (0.03-0.15) |  | 0.28 (0.09-0.84) |  |
| CCI Weight |  | 0.13 |  | 0.05 |
| 0 (reference) | 1.00 |  | 1.00 |  |
| ≥ 1 | 0.58 (0.28-1.18) |  | 2.47 (0.99-6.16) |  |
| Analyses only include patients with CCI data.  aOR = adjusted odds ratio; CCI = Charlson comorbidity index; CI = confidence interval; OR = odds ratio, TBSA = total body surface area. | | | | |

| **Supplementary Table 3. Predictors of deciding to withhold or withdraw active treatment within 24 hours** | | | | |
| --- | --- | --- | --- | --- |
|  | **OR (95% CI)** | ***p-*value** | **aOR (95% CI)** | ***p-*value** |
| Age | 0.98 (0.96-0.99) | < 0.001 | 1.02 (1.00-1.04) | 0.049 |
| Male |  | 0.08 |  | 0.73 |
| No (reference) | 1.00 |  | 1.00 |  |
| Yes | 1.58 (0.95-2.65) |  | 0.90 (0.45-1.74) |  |
| Flame burn |  | < 0.001 |  | 0.51 |
| No (reference) | 1.00 |  | 1.00 |  |
| Yes | 6.24 (2.89-13.50) |  | 1.44 (0.49-4.22) |  |
| Unintentional injury |  | 0.002 |  | 0.29 |
| No (reference) | 1.00 |  | 1.00 |  |
| Yes | 0.43 (0.26-0.73) |  | 1.46 (0.71-3.12) |  |
| %TBSA burned | 1.04 (1.03-1.05) | < 0.001 | 1.04 (1.03-1.06) | < 0.001 |
| Inhalation injury |  | < 0.001 |  | 0.03 |
| No (reference) | 1.00 |  | 1.00 |  |
| Yes | 4.38 (2.53-7.60) |  | 2.22 (1.06-4.63) |  |
| Full thickness burn |  | 0.002 |  | 0.97 |
| No (reference) | 1.00 |  | 1.00 |  |
| Yes | 3.38 (1.58-7.24) |  | 0.98 (0.36-2.67) |  |
| aOR = adjusted odds ratio; CI = confidence interval; OR = odds ratio; TBSA = total body surface area. | | | | |
